# Effects of the surface layer of steel samples after ball burnishing on friction and wear in dry reciprocating sliding

**DOI:** 10.1038/s41598-023-38534-7

**Published:** 2023-07-13

**Authors:** Slawomir Swirad, Andrzej Gradzik, Kamil Ochał, Pawel Pawlus

**Affiliations:** Rzeszow University of Technology, Powstancow Warszawy 8 Street, 35-959 Rzeszow, Poland

**Keywords:** Engineering, Materials science

## Abstract

The effects of ball burnishing on tribological behaviour in dry reciprocating motion have not yet been studied. This work attempts to fill this gap. The steel disc samples after milling were ball burnished. Due to ball burnishing, the average surface height decreased to 85% and the microhardness increased to 20%. Burnishing also generated the compressive residual stresses that were responsible factor to enhance the hardness of the steel surface. Trbological tests were carried out in reciprocating motion under dry sliding conditions. A 10 mm diameter ceramic ball from WC material contacted the steel disc. Ball burnishing was found to lead to improvements in disc wear and friction of the sliding pair. The maximum decreases in friction coefficient and wear volume compared to the milled sample were 39% and 85%, respectively. Samples of the lowest amplitude and high microhardness led to the highest behaviour.

## Introduction

Ball burnishing is a finishing process that causes plastic deformation of a surface in which a hard ball is moved and pressed onto the surface. It leads to an improvement in the surface finish as well as in the physical and mechanical properties of the workpiece. Because environmental pollution is small, this process is an interesting alternative to grinding. Typically, the diameter of the sphere is between 3 and 12 mm. In most of the works, the quality of the surface layer was estimated on the basis of surface roughness characterized by the Ra parameter (average roughness height), microhardness and residual stresses^1^. Typically, the burnishing load, forward speed, and feed were the input parameters^2^. Ball burnishing generally leads to surface smoothing, increased hardness, and compressive stresses^3^.

The researchers mainly studied the effects of ball burnishing on the decrease in the height of the roughness^4–11^.

Attabi et al. ^4^ found that ball burnishing of AISI 316L steel led to a decrease in the Ra parameter of up to 93%.

Banh et al.^5^ reached a decrease in the Ra parameter of oxygen-free copper from 0.89 (after milling) to 0.18 µm due to ball burnishing.

Dzionk et al.^6^ obtained a reduction in the Sa parameter (areal extension of the Ra parameter) of almost 50% due to ceramic ball burnishing of hardened shaft.

Jerez-Mesa et al.^7^ achieved a decrease in rms. roughness height Sq of Ti-6Al-4 V surfaces due to ball burnishing. Changes in skewness Ssk and kurtosis Sku were also considered. The effect of additional vibration on the reduction in surface height was positive when the initial value of the Sq parameter was less than 2 µm.

Vaishya et al.^8^ achieved a decrease in the height of the roughness of 95% of material used in optical moulds due to ball burnishing of tungsten carbide, the initial surface roughness determined by the Sa (Ra) parameter was 5 µm. An increase in the burnishing load led to surface smoothing.

Kanovic et al.^9^ obtained a decrease in the Ra parameter of the steel surface from 3.4–4.5 to 0.13 µm due to burnishing by a ceramic ball.

El-Tayeb et al.^10^ found that a decrease in ball diameter caused a reduction in the surface roughness height of aluminium alloy by 75%.

Swirad et al.^11^ achieved a decrease in the Sq parameter of the martensitic steel sample due to ball burnishing.

Effects of ball burnishing on hardness increase were also studied ^12–19^.

Hamadache et al.^12^ revealed that the surface layer hardening exponent of the surface layer increased by 10% due to the burnishing of the diamond ball of 36NiCrMo6 steel.

Bourieba et al.^13^ found that ball burnishing caused increases in hardness and rupture strength of the nearly 30% of samples of S355JR steel. In^14^ they achieved an increase in hardness of 45%.

Revenkar et al.^15^ obtained improvements in the height of the surface roughness and hardness of the titanium alloy of 77 and 17%, respectively, due to ball burnishing.

Buldum and Cagan^16^ achieved a reduction in the height of the surface roughness and increase in microhardness of the magnesium alloy due to ball burnishing.

Rodriguez et al.^17^ as well as Swirad and Pawlus^18,19^ revealed that a great burnishing load led to an increase in surface hardness; however, too high force may cause a growth in surface amplitude.

Effects of ball burnishing on residual stresses were also analysed^20–23^.

Chomienne et al.^20^ studied the effect of burnishing parameters on the residual stress profile and found that the normal force mostly influences the thickness of the affected layer.

Alshareef et al.^21^ studied the relation between burnishing factors and residual stresses of ball-burnished AISI 8620 steel. The burnishing pressure and the feed rate considerably affected the residual stresses.

Zhang et al.^22^ found that ball burnishing transformed tensile residual stresses after turning into compressive stresses.

Han et al.^23^ optimised the surface roughness height and residual stresses after ball burnishing.

Researchers studied the effect of burnishing on surface quality changing load, speed and feed^2,20–23^, Jerez-Mesa et al.^7^ changed feed and speed, El-Tayeb et al.^10^ burnishing speed and load, Swirad et al.^11^, Rodriguez et al.^17^ and Swirad and Pawlus^18,19^ burnishing force.

Surface topography is related to the tribological properties of the machine elements. It plays a significant role in the initial period of machine life, which is frequently associated with running-in period. However, the effect of surface topography can be extended to normal functioning of sliding pairs, especially for similar hardness and residual stresses of the surface layers^24^. The surface topography affected the tribological behaviours of sliding pairs in dry sliding^25–32^. 

Elwasli et al.^25^ tested an aluminum alloy plate with various surface topographies against ball bearing steel in reciprocating dry sliding. It was found that for rough disc surfaces and especially smooth disc surfaces, friction and disc wear levels were higher when the mean profile slope was higher.

Menezes et al.^26^ studied the steel plate effect of the surface topography on the coaction with lead pin. In dry conditions, the coefficient of friction was strongly correlated with the profile slope of the steel plate.

The disc of WC-CoCr cast iron was coacted with a commercial pin from a low-metallic friction material in dry unidirectional sliding^27^. Pin wear increased with increasing disc roughness height, contrary to friction, which was higher for the smooth disc surface. The wear of the disc was marginal.

Wen et al.^28^ tested the co-action between a steel disc of smaller hardness and a steel ball of higher hardness under dry unidirectional sliding. Discs were machined by grinding, milling, and turning, therefore, they had different surface topographies. The surface topography of the discs was found to affect the coefficient of friction only during running-in. The smoother surface led to higher friction in running-in, however, it quicker obtained stable friction.

Dzierwa et al.^29^ found that the higher surface roughness of a steel disc led to a longer distance to obtain steady state conditions in dry unidirectional sliding tests. A larger disc roughness led to lower volumetric wear.

Khun et al.^30^ obtained a smaller friction and wear of the niobium samples with increasing roughness height in dry friction tests in ball-on-disc configuration.

Zhu and Huang^31^ performed dry tests between steel samples of different surface topographies and polished ball from corundum ceramic. They found that for similar skewness and kurtosis an increase in roughness height was observed.

In^32^ the block of composite material coacted with the steel ring under dry friction conditions. The block wear level was generally higher when the roughness height of the ring counter-sample was higher.

The experiments were carried out in pin-on-disc configuration under dry friction conditions^33^. Both the pin and the discs were made from steel. The surface height, determined by the Sq parameter, decreased as the test progressed. The Sq parameter and rms. slope Sdq were positively correlated with the coefficient of friction.

Hardness is considered a primary material property that defines wear resistance^34^. The harder surface led to wear reduction^35–38^.

Jeong et al.^35^ found that abrasive wear resistance was linearly proportional to the hardness of the coatings.

Jiang et al.^36^ revealed that when adhesive-predominant wear appeared, the wear resistance of the steel sample was proportional to the hardness in dry sliding tests.

Mendikos et al.^37^ obtained a smaller wear of harder composite resins.

Due to hardness increase, the wear reduction in dry sliding was achieved in ^38^ under dry contact conditions.

Compressive residual stresses contribute to the prolongation of the fatigue life of machine elements ^38^. They also affected positively wear resistance^39–43^.

Luo et al.^39,40^ found that the compressive stresses in the HVOF-sprayed WC–Co coatings had significant positive effects on wear resistance differently from tensile stresses.

Liu et al.^41^ revealed that high compressive residual stresses could improve the wear resistance of induction-hardened medium carbon steel.

Dancer et al.^42^ obtained an increase in the wear resistance of severe wear of alumina–silicon carbide two-layered composites due to the presence of compressive residual stresses.

Alanazi et al.^43^ achieved a wear resistance increase of nanocrystalline coatings due to an increase in compressive residual stresses only up to a certain stress value.

The tribological effects of ball burnishing were studied^4,10,18,44,45^.

Ball burnishing caused the improvement in fatigue behaviour for samples subjected to rotational fatigue tests ^1^.

Attabi et al.^4^ achieved due to ball burnishing wear reduction in dry friction up to 53.4% in unidirectional sliding in ball-on-disc configuration. However, a decrease in the friction force was obtained only for the smoothest burnished surface of the workpiece.

El-Tayeb et al.^10^ obtained a reduction in friction and wear in dry sliding tests due to ball burnishing, in comparison to the turned specimen.

Swirad and Pawlus^18^ reduced friction and wear due to ball burnishing in unidirectional dry sliding, compared to milled surface.

Ball burnishing of steel resulted in increasing hardness of 31% and a decrease in the Ra parameter of 80% leading to a reduction in impact wear volume between 53 and 62%^44^.

Revankar^45^ achieved due to ball burnishing a decrease in the wear rate by 52%, and the friction coefficient by 64% of titanium alloy in unidirectional dry tests compared to the turned surface.

One can see from the analysis of literature that the results of studies concerning the effects of surface texture height on friction under dry sliding conditions were sometimes contradictory. Typically, increases in hardness and presence of residual stresses improved wear resistance of the sliding elements. Most tribological tests under the dry friction regime were carried out in unidirectional sliding. The reciprocating test is one of the commonly used laboratory wear testing methods to determine the wear behaviour of engineering materials, used in components whose normal operation result in periodic reversals in the direction of relative sliding. However, dry tests in reciprocating motion were seldom carried out. Specifically, the authors of this paper did not find results of the effect of ball burnishing on friction and wear in dry reciprocating tests. This work attempts to fill this gap. The study of join effects of surface topography, hardness, and residual stresses discs after ball burnishing on friction and wear in dry reciprocating sliding is the novelty of this work. Ball-on-flat reciprocating tests are functionally important because they simulate the co-actions of rubbing components whose normal operations result in periodic reversals in the sliding direction.

## Materials and methods

The investigations were performed in ball-on-disc configuration in a dry reciprocating motion. Ceramic ball of 10 mm diameter made from WC material was placed in contact with disc of 25 mm diameter made of 42CrMo4 steel of 44 ± 2 HRC hardness. The tests were carried out using the Optimol SRV5 tribotester at a temperature of 30 °C, the relative humidity was 40–50%, the frequency was set to 50 Hz, the stroke was set to 1 mm and the number of cycles was set to 45,000. The following normal forces were applied: 20, 40 and 60 N. The number of test repetitions was three. Table 1 presents the test parameters. The normal loads and frequency of oscillation were similar to those used in previous research under fretting gross conditions by the authors of this paper ^19^. The working parameters ensured operating in dry reciprocating sliding, not fretting. Three normal loads were selected to study the tribological effect of ball burnishing at various conditions.

**Table 1:** Reciprocating sliding parameters.

Parameter	Value
Frequency	50 Hz
Humidity	40–50%
Stroke	1 mm
Normal load	20, 40, 60 N
Number of cycle	45,000

**Figure 1:**
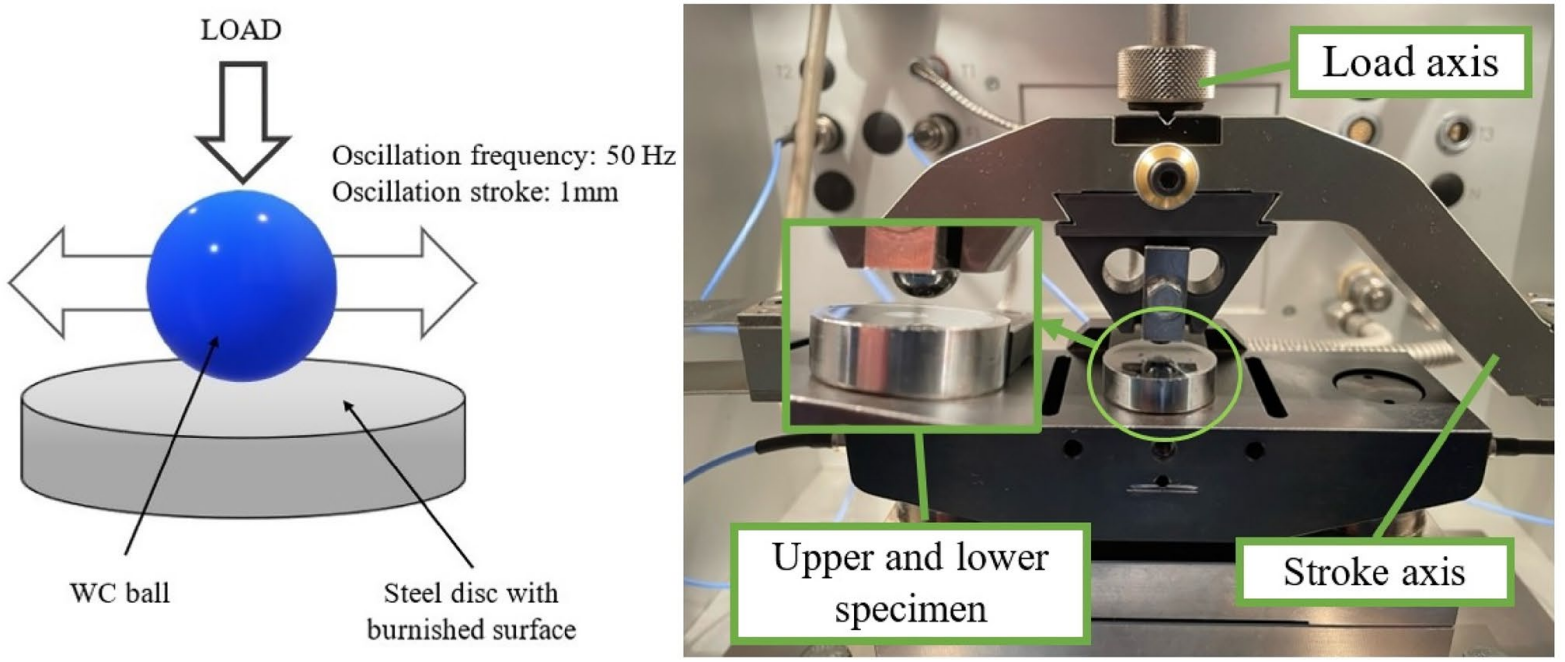
The scheme of the tribotester.

Figure 1 presents the tribotester scheme.

Before tribological tests, the disc samples after milling were ball burnished with the application of the Haas CNC Vertical Mill Center VF-1. Table 2 presents the burnishing parameters. The burnishing pressures used comprise the range of available possibilities.

**Table 2:** Burnishing parameters.

Parameter	Value
Burnishing strategy	Spiral
Speed	650 mm/min
Ball	6 mm
Pressure	10, 20, 30, 40 MPa

Before and after tribological tests, the surface topographies of the sliding elements were measured using the Talysurf CCI Lite white light interferometer with 0.01 nm vertical resolution. The measured surfaces of the discs were only levelled, and digital filtration was not used. Before calculations of the parameters, spikes were removed, and non-measured points were filled.

**Figure 2:**
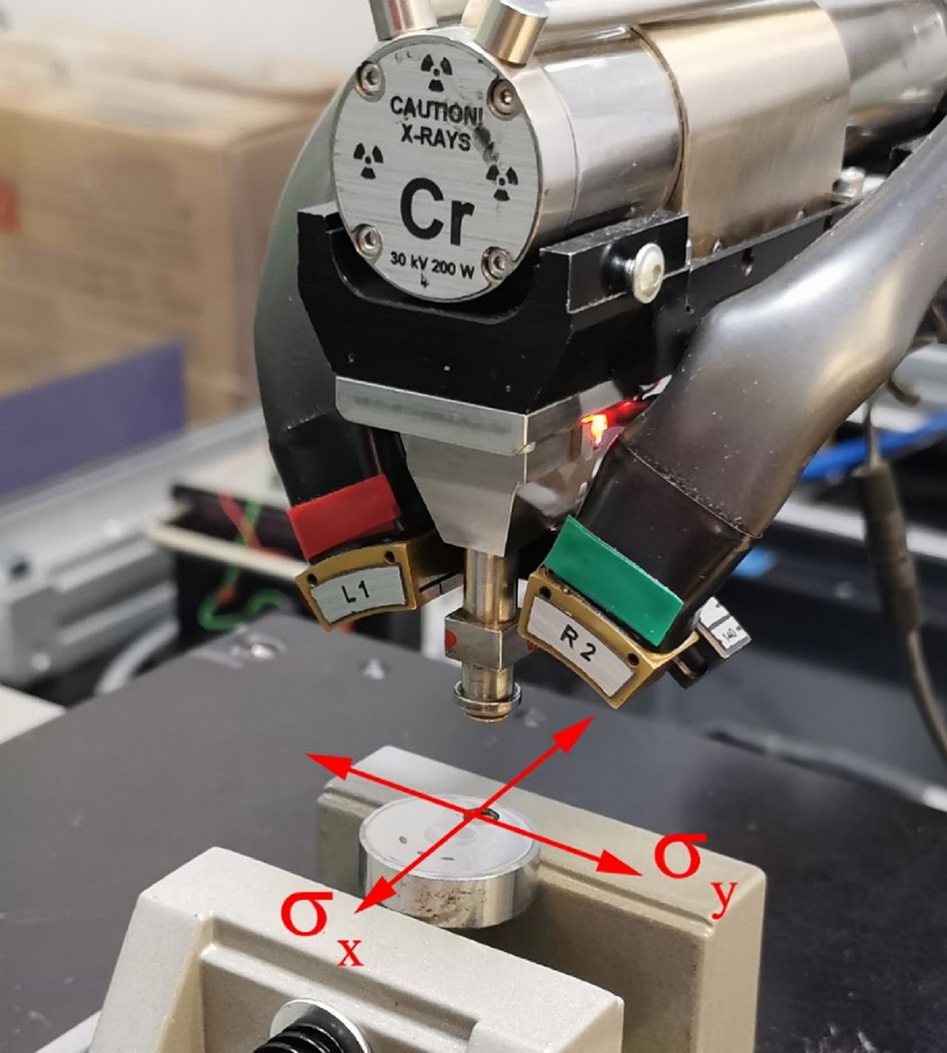
Scheme of residual stress measurements.

Before tribologcal tests, residual stress measurements were caried out by X-ray diffraction using a PROTO iXRD Combo (Proto Manufacturing Ltd., Oldcastle, Canada) X-ray diffractometer.. The sin^2^ψ method and the ω geometry were applied according to the EN 15305:2008 standard. An X-ray tube with Cr anode was used as a radiation source (λ_CrKα_ = 0,2291 nm) and a round aperture with 1.00 mm diameter. The diffraction peaks of the ferrite lattice plane{211} were used in the measurements and the Bragg angle 2θ = 156.00°. The following ψ angle values were used: ± 37.00°, ± 32.60°, ± 27.85°, ± 23.80°, ± 15.74°, ± 13.00°, ± 12.00°, ± 8.60°, ± 8.26°, ± 3.85° and ± 0.20°. The diffraction peak positions were analysed using the Gaussian peak fit function. X-ray elastic constants used in the research were: 1/2S_2_ = 5.92 × 10^−6^ MPa and − S_1_ = 1.27 × 10^−6^ MPa. The rest of the measurement conditions were as follows: exposure time − 2 s, number of exposures per ψ angle − 10, gain material β-Ti. Measurements were carried out in two perpendicular directions (Fig. 2). The residual stress gradient was determined using measurements at the following depths below the surface of the burnished zone: 25, 50, 75, 100, 150, 200, 300, 400, 500, 600, 700 and 800 μm. The removal of the surface layer for the residual stress gradient measurements was carried out by electrolytic polishing using Proto 8818-V3 electrolytic polisher (Proto Manufacturing Ltd., Oldcastle, Canada).

Surface hardness was measured using Reicherter Brivisor KL2 Vickers micro-indenters.

After tribological tests, SEM images were acquired with the Phenom ProX desktop scanning electron microscope equipped with a thermionic CeB_6_ (cerium hexaboride) source and a high sensitivity multi-mode backscatter detector (BSD). SEM measurement was performed at a voltage of 10 kV, the imaging magnifications were commonly fixed around 200 and 2100 times. The analysis of the worn surface using profilometers and SEM is common^46–48^.

## Results and discussion

Table 3 presents the results of the surface texture measurements of the discs prior to tribological tests. Parameters from the ISO ISO 25178:2 standard were analysed^49^. The milled surface had a deterministic character, the Sku kurtosis was smaller than 3 and the texture aspect ratio Str was small (0.03). After ball burnishing, the amplitude parameters decreased: rms. height Sq, arithmetical average height Sa, maximum peak height Sp, maximum valley depth Sv, maximum height Sz; rms. Sdq slope and average peak curvature Spc also decreased. The surface height decreased when the burnishing pressure increased from 10 to 30 MPa and increased for the highest burnishing pressure. The skewness Ssk decreased due to ball burnishing and obtained the smallest negative value for the lowest burnishing pressure. Sku kurtosis increased and obtained values similar to 3, which proved that the content of the random component increased due to the ball burnishing process. The smallest decrease in the parameters Sdq and Spc occurred for the lowest burnishing pressure. The parameters from the Sk family: core roughness depth Sk and reduced peak height Spk decreased due to burnishing, the relative changes in the Spk parameter were higher. The reduced valley depth Svk increased for burnishing pressures of 10 and 20 MPa and decreased for higher pressures. These results proved of higher changes in the the surface texture in peak and core parts compared to the valley portion. The parameters from the V family: core material volume Vmc, peak material volume Vmp, core void volume Vvc and dale void volume Vvv decreased. The Str parameter increased due to the burnishing up to 0.74, burnishing pressure was 30 MPa. This surface had an isotropic character. The correlation length Sal and the texture aspect ratio Str increased as a result of burnishing. Peak density Spd decreased as the burnishing pressure increased. A decrease in the height of the roughness due to ball burnishing was reported in the technical literature^4–11,15,16^.

**Table 3:** Results of the surface texture parameter measurements.

Parameters		Milled	Burnished			
			10 MPa	20 MPa	30 MPa	40 MPa
Sq	µm	0.8719	0.3266	0.2576	0.1383	0.2026
Ssk		0.4256	− 0.254	− 0.0286	− 0.0126	− 0.002
Sku		2.151	3.365	2.983	2.547	3.071
Sp	µm	2.765	1.026	0.8021	0.3274	0.488
Sv	µm	1.934	1.262	0.772	0.3246	0.4818
Sz	µm	4.699	2.289	1.574	0.6519	0.9698
Sa	µm	0.7429	0.2568	0.2041	0.1122	0.1626
Sk	µm	1.966	0.7969	0.6413	0.3696	0.5188
Spk	µm	0.9994	0.3019	0.2527	0.1139	0.1916
Svk	µm	0.2415	0.3807	0.2639	0.1168	0.1808
Sal	mm	0.04381	0.1298	0.1956	0.1444	0.1623
Str		0.03289	0.4127	0.1752	0.7419	0.1808
Sdq		0.06453	0.03792	0.00770	0.01033	0.00952
Spd	1/mm^2^	234.7	602.5	516.5	296.8	111.81
Spc	1/mm	49.04	34.39	4.161	5.894	4.050
Vmp	µm ^3^/ µm ^2^	0.03	0.015	0.0124	0.0057	0.00919
Vmc	µm ^3^/ µm ^2^	0.8179	0.2915	0.2263	0.1298	0.1844
Vvc	µm ^3^/ µm ^2^	1.266	0.3679	0.3112	0.1732	0.2575
Vvv	µm ^3^/ µm ^2^	0.03	0.015	0.0124	0.0057	0.00919

**Figure 3:**
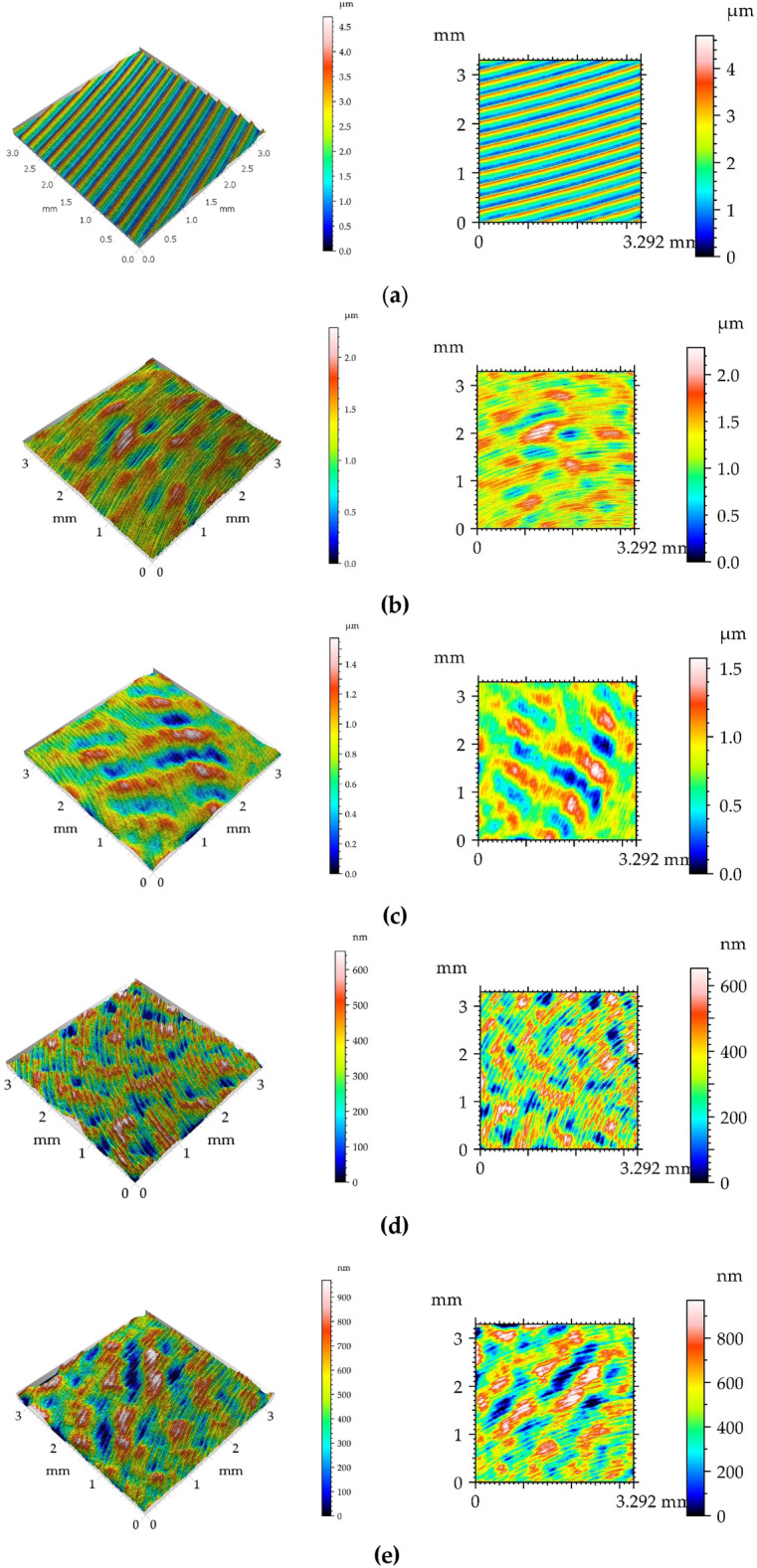
3D views (left) and pseudo-colour images (right) of the milled disc surface (**a**) and of burnished disc surfaces with pressures of 10 (**b**), 20 (**c**), 30 (**d**) and 40 MPa (**e**).

**Figure 4:**
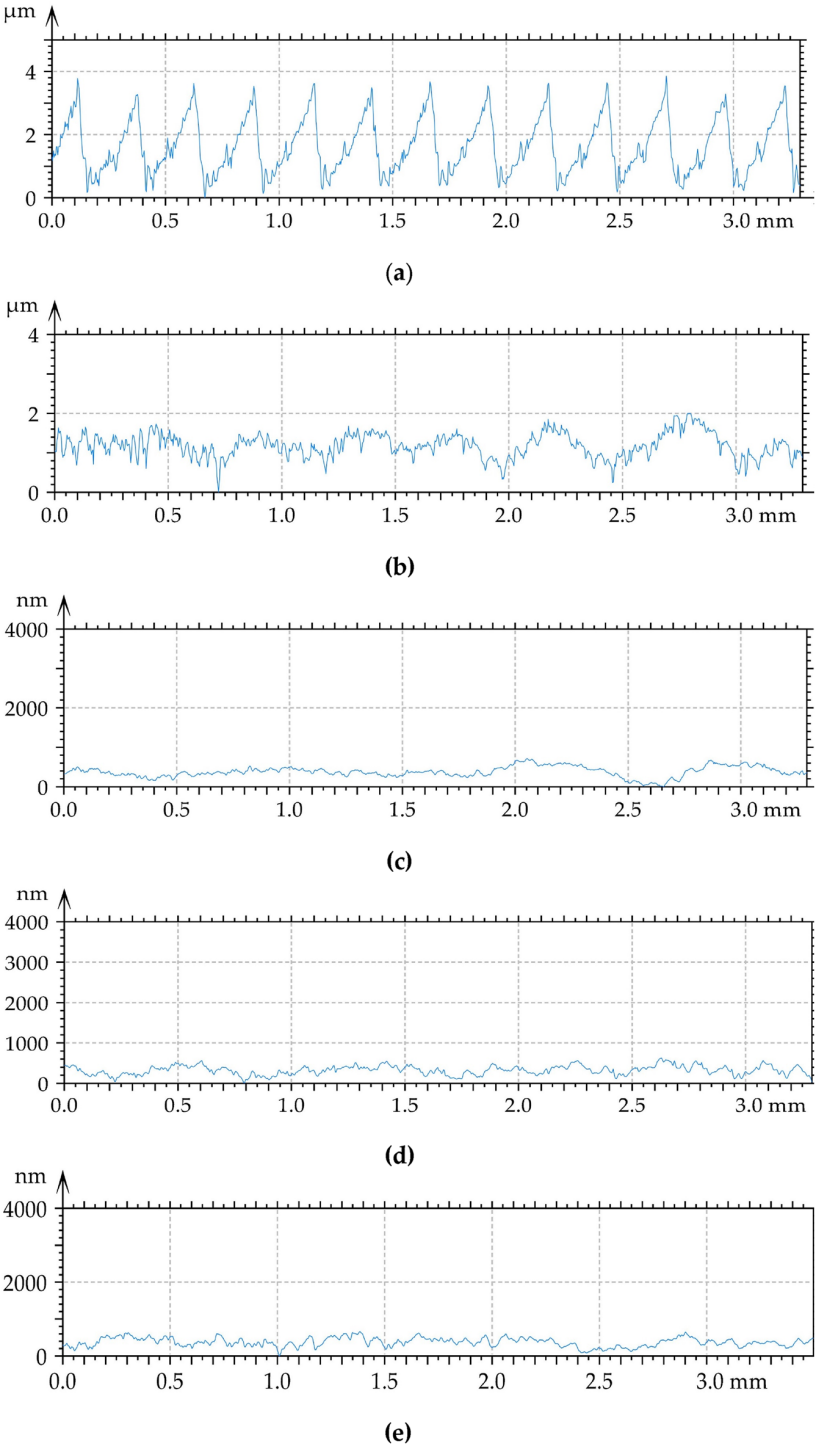
Typical profiles of the disc surface after milling (**a**) and of burnished surfaces with the following pressures: (**b**), 20 (**c**), 30 (**d**), and 40 MPa (**e**).

Figure 3 presents pseudo-colour images and isometric views of the surface analysed prior to tribological tests. Figure 4 presents typical profiles of the machined surfaces. One can see from the analysis of Figs. 3 and 4 that the milled surface had a periodic character with a small amount of random component. Due to ball burnishing with pressures of 20, 30 and 40 MPa, profile heights decreased and surface textures obtained had random character. Periodic component was visible on surface after ball burnishing with the smallest pressure of 10 MPa.

**Figure 5:**
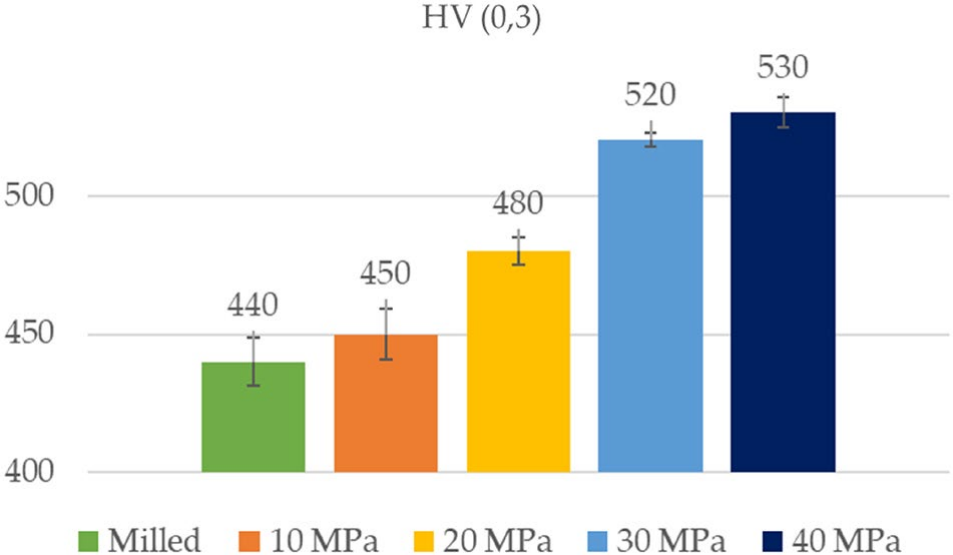
Microhardness values of machined disc samples.

Figure 5 presents the result of microhardness measurements of machined surfaces. Ball burnishing led to an increase in microhardness. Ball burnishing operate by surface plastic deformation and without removal of material. This results in a new surface integrity characterised by a flattening of the roughness profile and a hardening of superficial layers^13,14^. An increase in burnishing pressure caused increase in hardness^17–19^ The increase was marginal when the smallest burnishing pressure of 10 MPa was applied. Burnishing pressure of 20 MPa caused an increase in microhardness of almost 9%. The application of pressures of 30 and 40 MPa increased the microhardness of about 18 and 20%, respectively.

**Figure 6:**
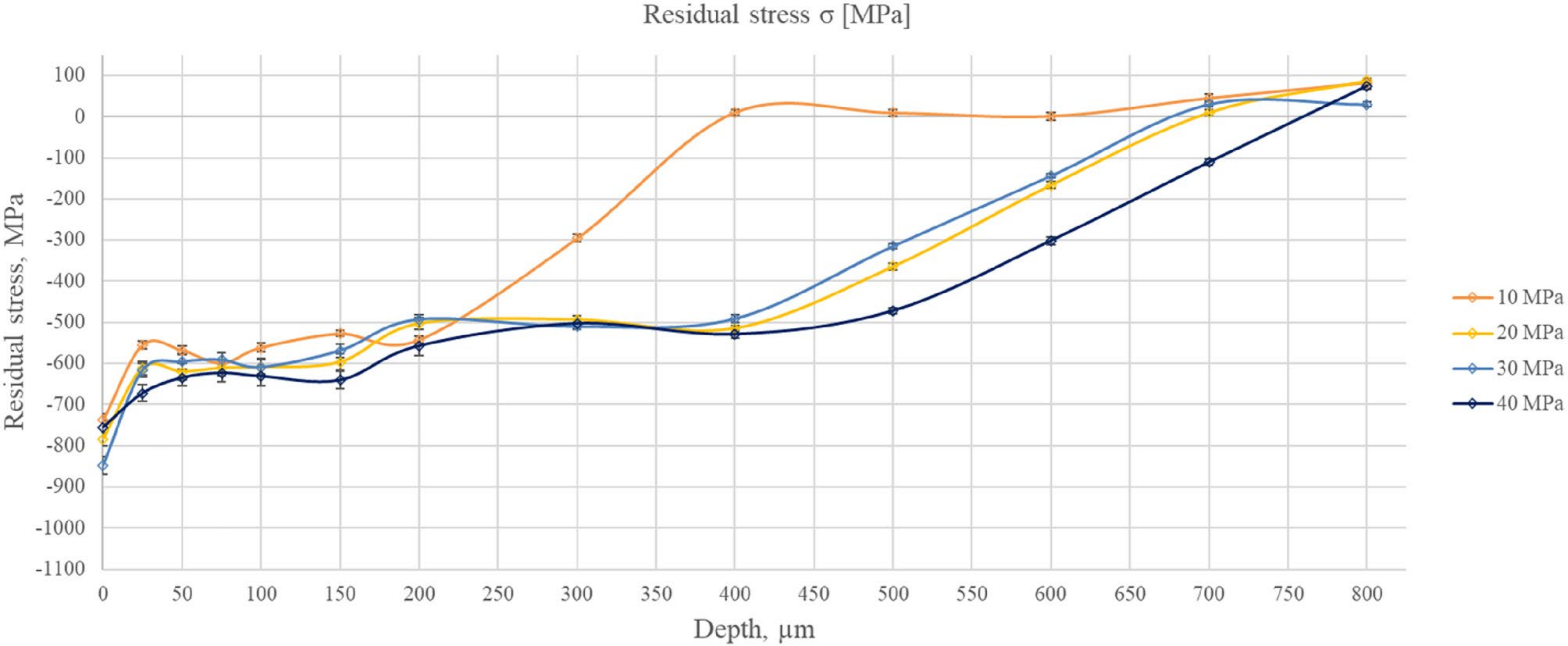
Average residual stress σ distributions versus material depth for burnished samples.

The results of the residual stress measurement of burnished samples are presented in Table 4. Figure 6 presents the average residual stress distributions versus the depth of the material. For the milled workpiece, the average residual stress was 383 ± 13 MPa.

**Table 4:** Results of residual stress measurements.

Burnishing Pressure												
Depth [µm]	10 MPa			20 MPa			30 MPa			40 MPa		
	σ_x_ MPa	σ_y_ MPa	σ MPa	σ_x_ MPa	σ_y_ MPa	σ MPa	σ_x_ MPa	σ_y_ MPa	σ MPa	σ_x_ MPa	σ_y_ MPa	σ MPa
0	− 1012	− 463	− 738 (± 16)	− 1003	− 565	− 784 (± 16)	− 1068	− 627	− 848 (± 21)	− 974	− 536	− 755 (± 16)
25	− 737	− 373	− 555 (± 9)	− 804	− 419	− 612 (± 17)	− 808	− 424	− 616 (± 17)	− 895	− 449	− 672 (± 21)
50	− 742	− 394	− 568 (± 10)	− 844	− 396	− 620 (± 20)	− 806	− 385	− 596 (± 19)	− 869	− 400	− 635 (± 21)
75	− 770	− 428	− 599 (± 10)	− 851	− 371	− 611 (± 20)	− 832	− 350	− 591 (± 18)	− 862	− 384	− 623 (± 21)
100	− 706	− 416	− 561 (± 10)	− 848	− 370	− 609 (± 21)	− 845	− 372	− 609 (± 18)	− 882	− 380	− 631 (± 23)
150	− 589	− 466	− 528 (± 7)	− 787	− 406	− 597 (± 21)	− 743	− 394	− 569 (± 27)	− 865	− 416	− 641 (± 21)
200	− 563	− 522	− 543 (± 8)	− 632	− 374	− 503 (± 15)	− 620	− 365	− 493 (± 11)	− 717	− 397	− 557 (± 24)
300	− 329	− 263	− 296 (± 10)	− 544	− 443	− 494 (± 9)	− 552	− 468	− 510 (± 7)	− 612	− 393	− 503 (± 10)
400	− 1	21	10 (± 8)	− 522	− 507	− 515 (± 8)	− 503	− 479	− 491 (± 9)	− 582	− 476	− 529 (± 10)
500	3	22	13 (± 8)	− 352	− 377	− 365 (± 8)	− 313	− 316	− 315 (± 6)	− 463	− 481	− 472 (± 7)
600	8	− 6	1 (± 10)	− 173	− 160	− 167 (± 8)	− 152	− 135	− 144 (± 6)	− 312	− 291	− 302 (± 10)
700	54	36	45 (± 9)	− 56	76	10 (± 7)	19	42	31 (± 8)	− 127	− 95	− 111 (± 7)
800	75	89	82 (± 7)	74	100	87 (± 7)	26	34	30 (± 7)	72	76	74 (± 8)

Due to ball burnishing, the tensile residual stresses were transformed into compressive residual stresses. The burnishing process generated the compressive residual stresses in both perpendicular directions σ_x_ and σ_y_, which were responsible factors to enhance the surface hardness of the steel. The presence of residual compressive stresses in the surface layer was also found in^20–23^. The thickness of the affected layer was the smallest (0.4 mm) for the lowest burnishing pressure of 10 MPa. In other cases, this thickness was 0.7–0.8 mm. Chomienne et al.^20^ also revealed that the normal force mostly influences the thickness of this layer. For depths smaller than 75 µm burnishing with the smallest pressure led to the lowest compressive stress however, burnishing with the highest pressure caused the highest stress. Burnishing pressure affected residual stresses also in^21,22^.

**Figure 7:**
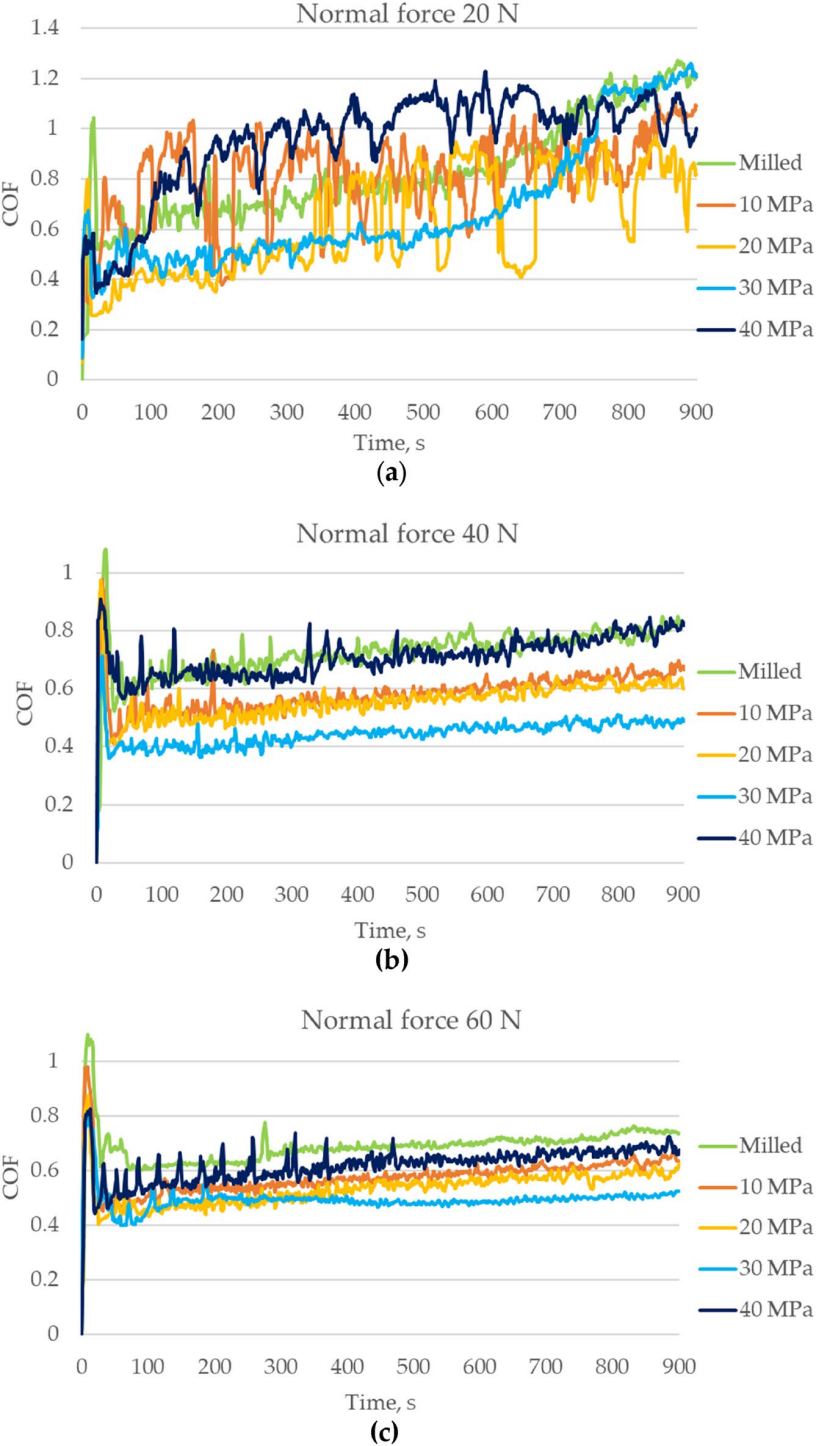
The friction coefficient as a function of time, for the normal force of 20 (**a**), 40 (**b**) and 60 N (**c**).

Figure 7 shows the changes in the friction coefficients versus time for various normal loads. Initial fluctuations in friction force occurred during the first 10 s. Then the friction force increased. This increase was the highest for the lowest normal load, approximately 1.7 times; for higher loads, the friction force increased 1.2 times as the test progressed. The friction coefficient decreased when the normal force increased. The lowest normal force was associated with the greatest friction variation. In this case, the highest friction force took place for assembly of the burnished sample with the highest pressure; however, the burnishing pressure of 20 MPa caused the lowest friction force. In the initial test part, a low value of the friction coefficient was obtained for the disc sample after burnishing with the pressure of 30 MPa; however, in the finishing test period, this coefficient increased dramatically. When the normal force increased to 40 N, the burnishing pressure of 30 MPa led to the smallest, while the pressure of 40 MPa and the milled sample led to the highest friction force. When the normal load was the highest, the disc specimen after milling corresponded to the highest friction force, followed by burnished samples created by pressures of 40, 10, 20 and 30 MPa. In the last case, the friction coefficient was stable after 200 s.

**Figure 8:**
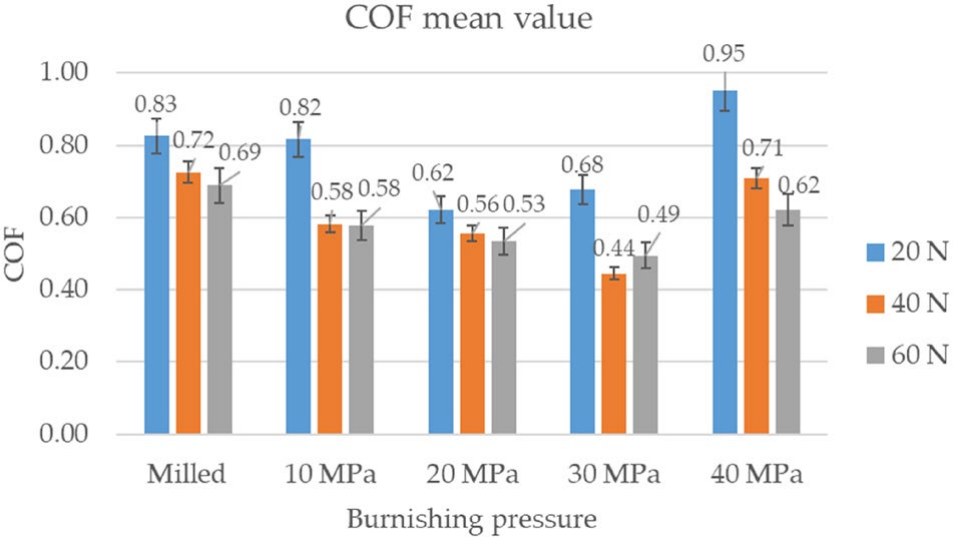
The average coefficients of friction.

Figure 8 presents the mean values and error bars of the coefficient of friction of various sliding pairs and operating conditions. For the lowest normal load of 20 N, the lowest coefficient of friction occurred for assemblies having burnished discs with pressures of 20 and 30 MPa. The highest burnishing pressure led to the highest coefficient of friction. When the medium normal load of 40 N was applied, the smallest coefficient of friction was achieved for sliding pairs with burnished samples with pressure of 30 MPa, followed by pressures of 20 and 10 MPa. The disc sample after milling and the burnished one with the maximum pressure corresponded to the highest coefficients of friction. When the highest normal load of 60 N was applied, the largest friction force occurred for the milled disc sample. Among assemblies with burnished discs, the smallest friction coefficient corresponded to a burnishing pressure of 30 MPa, followed by 20, 10 and then 40 MPa.

Generally, burnishing with a pressure of 30 MPa ensured the lowest frictional resistance. This behaviour is probably related to the smallest initial roughness height. A decrease in the coefficient of friction due to surface smoothing was also found in^18,19,33^. The highest friction forces were obtained for assemblies with a disc after milling and with a burnished disc with the greatest pressure.

**Figure 9:**
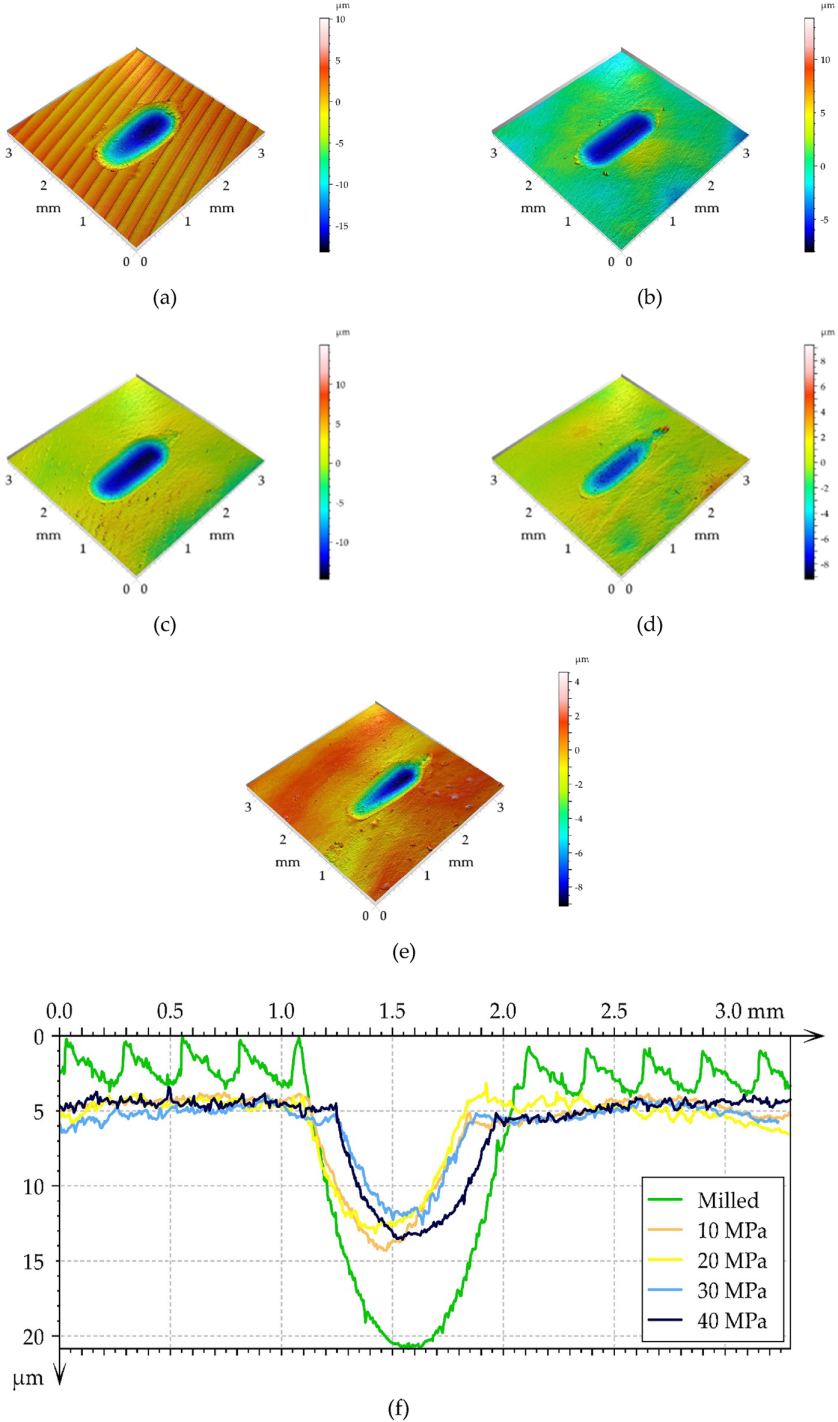
Isometric views of the surfaces of worn discs with a normal force of 20 N, (**a**) milled disc, (**b**) burnished discs with a pressure of 10 MPa, (**c**) 20 MPa, (**d**) 30 MPa, (**e**) 40 MPa and a series of profiles perpendicular to the wear scars (**f**).

**Figure 10:**
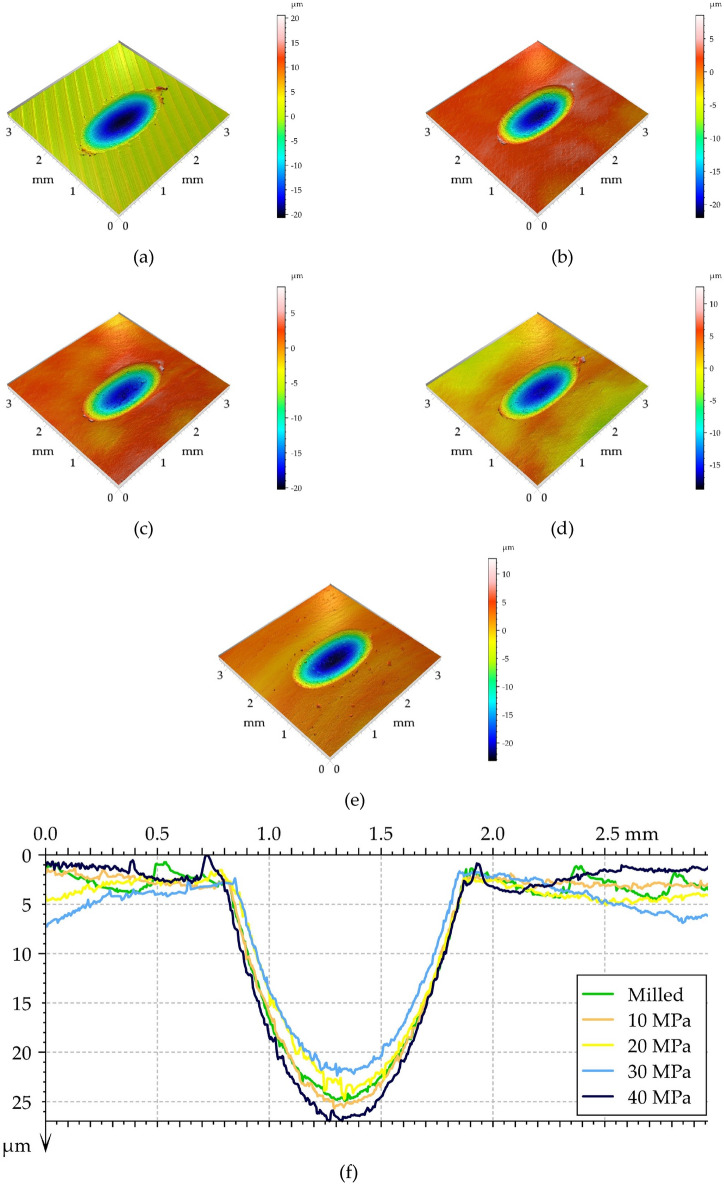
Isometric views of the surfaces of worn discs at a normal load of 40 N, (**a**) milled disc, (**b**) burnished discs for a pressure of 10 MPa, (**c**) 20 MPa, (**d**) 30 MPa, (**e**) 40 MPa and series of profiles perpendicular to the wear scars (**f**).

Figure 9 presents isometric views of the worn disc surfaces for the smallest normal force, including wear scars. Profiles perpendicular to wear scars are also shown. The analysis of Fig. 9 that abrasive wear mainly occurred. Plastic deformation also occurred, especially in the milled sample. The depth of the wear scar was low for the burnishing disc samples and high for the milled disc sample. The greater depth corresponded to the larger width of the wear scar, which was close to 1 mm for the milled disc. For other disc samples, the widths of the wear scars were smaller (near 0.75 mm). The depth of the largest wear scar was more than that of 2 times that of the smallest scar (Fig. 10f). For burnished samples, the lowest depth of wear scar occurred for pressure of 30 MPa.

Figure 10 shows isometric views of the worn disc surfaces for a medium normal force and profiles perpendicular to the wear scars. A growth in normal force resulted in increases in the depth of the wear scars. In each case, the width of the wear scar was similar to 1 mm. Again, the wear of the disc was abrasive with the possibility of plastic deformation (mainly in the milled sample). The smallest depth of wear scar was close to 20, while the highest was close to 25 µm, so the effect of machining on wear was smaller compared to the smallest load used. One can see from the analysis of Fig. 10f that the smallest depth of the wear scar corresponded to a burnished sample with the pressure of 30 MPa.

**Figure 11:**
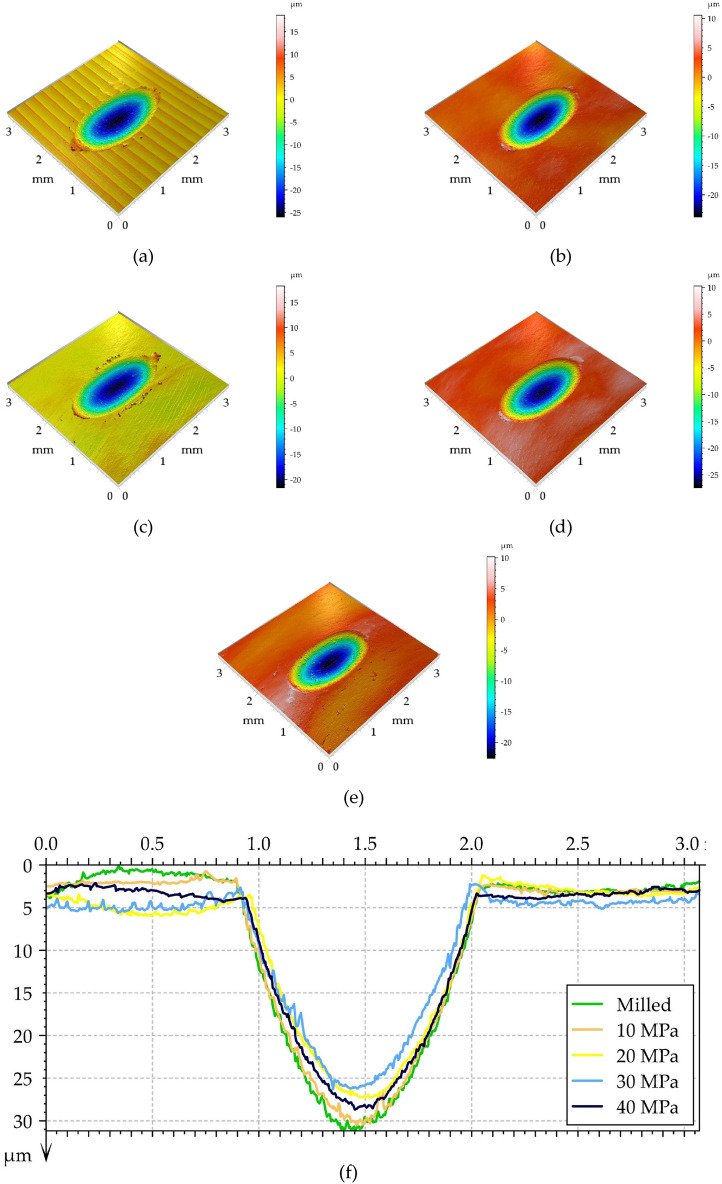
Isometric views of the surfaces of worn discs at a normal load of 60 N, (**a**) milled disc, (**b**) burnished discs for a pressure of 10 MPa, (**c**) 20 MPa, (**d**) 30 MPa, (**e**) 40 MPa and a series of profiles perpendicular to the wear scars (**f**).

Figure 11 presents isometric views of the worn disc surfaces for the highest normal force with wear scars. Profiles orthogonal to the wear scars are also shown. The wear of the disc was abrasive and plastic deformation also occurred. The depths of the wear scars were between 23 and 28 µm. Increasing the normal force from 40 to 60 N led to a growth in the wear depth.. The effect of the machining process on wear was smaller than that for the lowest normal load. One can see from the analysis of Fig. 11f that the smallest depth of wear scar was obtained for burnished samples, starting from burnishing pressure of 30 MPa, followed by 20, 40 and 10 MPa. Milling led to the maximum depth of the wear scar.

**Figure 12:**
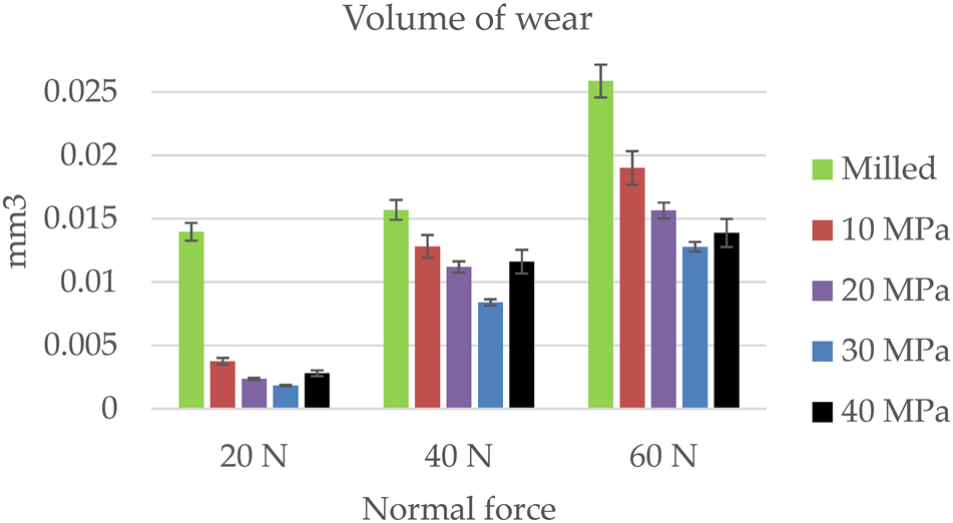
Wear volumes of discs.

Figure 12 presents the mean values of the wear volumes of the disc surfaces. Error bars (standard deviations) are also shown. An increase in normal load caused an increase in volumetric wear. Independently of normal load, the milled disc corresponded to the greatest volumes of wear. Among the burnished samples, the highest wear occurred for the smallest burnishing pressure. In all cases burnishing pressure of 30 MPa led to the smallest wear volumes. Burnishing pressures of 20 and 40 MPa corresponded to similar wear for the lowest and medium normal load. When the burnished samples were tested, the smallest wear variations due to test repetitions were found for the smallest applied load. The effect of the machining process on wear volume was the most important for the smallest normal load, when the ratio of the largest to the lowest wear was the largest, followed by the highest and medium normal loads. The wear levels of balls were negligible.

One can see from the analysis of Table 3 that ball burnishing reduced surface height when the pressure increased from 10 to 30 MPa. However, when the ball burnishing pressure increased to 40 MPa, the roughness height increased, compared to the pressure of 30 MPa. The roughness height that corresponded to the burnishing pressure of 40 MPa, determined by the amplitude parameters Sa, Sq, Sp, Sv, Sz, as well as parameters Spk, Svk, Sk and Vmp, Vvc, Vvv and Vmc, was smaller than that obtained for the pressure of 20 MPa. The surface obtained with the highest burnishing pressure was not homogeneous with the lowest peak density Spd, which could be related to poor tribological properties of the sliding pair. The growth in burnishing pressure resulted in an increase in microhardness. This increase was greatest for pressures of 30 and 40 MPa. However, changing the burnishing pressure from 30 to 40 MPa caused negligible increase in microhardness. Perhaps the burnishing pressure of 40 MPa was too high because it led to an increase in the amplitude of the roughness. Similar results were obtained in^17–19^. When the tendency to adhesion is small, the coefficient of friction should be higher for a rougher surface^49,50^. Therefore, one can predict the best tribological properties for the disc burnished with a pressure of 30 MPa (the smallest roughness height, high microhardness, and high compressive residual stresses). Due to the high roughness, small microhardness and tensile residual stresses, poor tribological properties of the sliding pair with milled disc sample can be predicted.

Similar friction trends were found for the medium and highest normal forces (Fig. 7). The lowest friction coefficients were achieved for a burnishing pressure of 30 MPa. The lowest roughness height was the most probable reason for it. The highest friction coefficients were obtained for sliding pairs with the disc after milling, and then the burnished disc with the highest pressure. The high friction resistance of the assembly with the milled disc was caused by the large amplitude of the surface texture. The non-homogeneous character of the disc burnished with the biggest pressure probably caused the high friction coefficient. These disc samples also led to high coefficients of friction for the smallest normal load applied. A large coefficient of friction was found for the burnished sample with the smallest pressure. Burnishing pressure of 30 MPa resulted in a relatively low friction coefficient. However, the smallest friction was reached for the burnished sample with a pressure of 20 MPa. Increasing the normal load caused the reduction of the coefficient of friction. This behaviour was caused by better accommodation of sliding surfaces as the load increased. In general, the surface texture of the disc sample seems to govern the frictional resistance. A decrease in friction force due to smoothing surface texture was also found in^18,19,33^.

**Figure 13:**
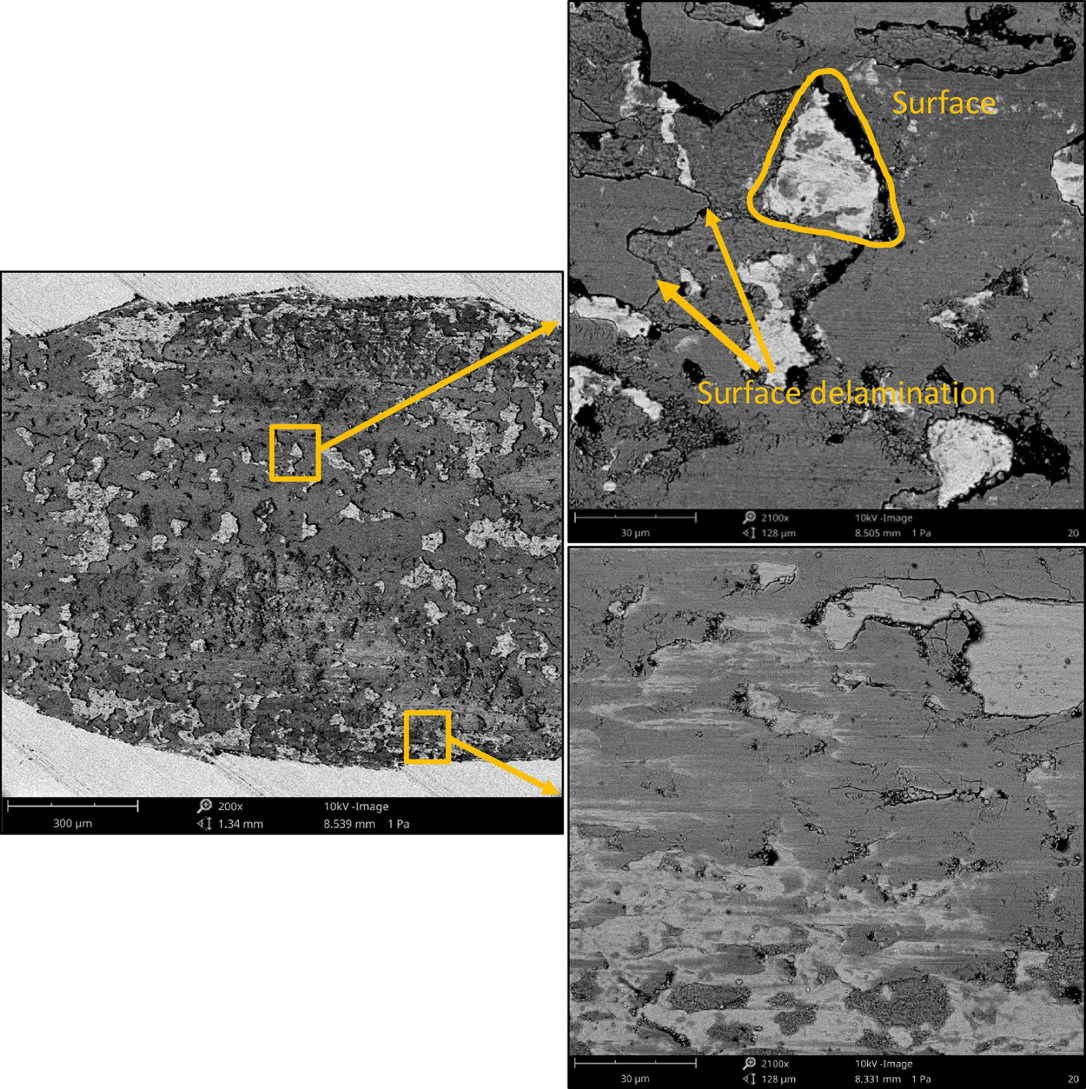
SEM images of the surface layer of the milled disc sample after the tribological test at the normal load of 60 N.

**Figure 14:**
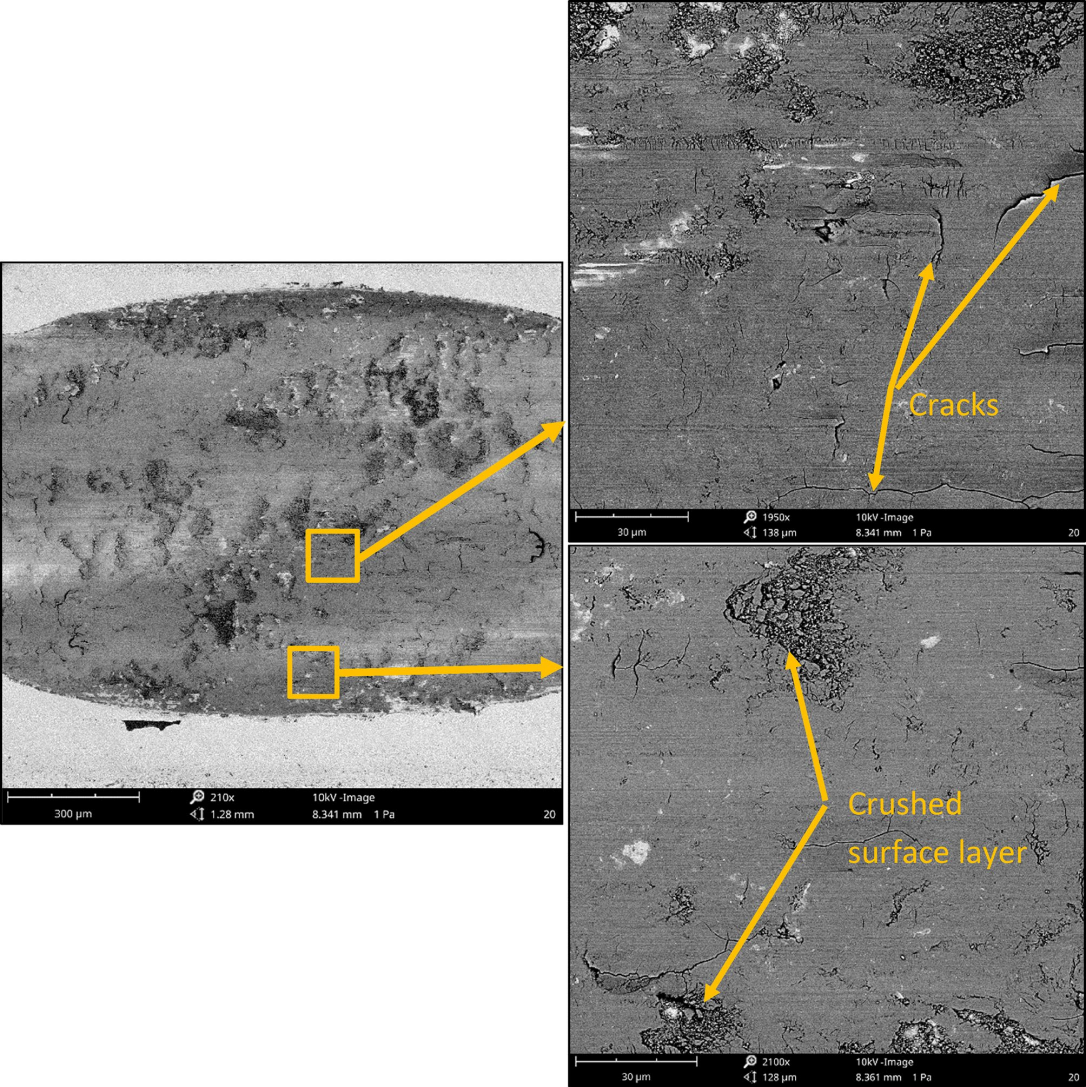
SEM images of the surface layer of the burnished disc sample with a pressure of 30 MPa after the tribological test at the normal load of 60 N.

One can see from the analysis of Figs. 9, 10 and 11 that disc wear had a mainly abrasive character. The ‘U shape’ ^51^ shows that the wear is non-adhesive. Plastic deformation also occurred, especially in the milled sample. The wear mechanisms are generally explained by using SEM images of worn surfaces (Figs. 13 and 14). On the image of worn milled disc sample (Fig. 14) one can see numerous debris and craters formation. The subsurface layer resulting from detachment of the outer layer is visible. Debris can be an additional factor increasing the wear process. Delamination of the surface layer occurred. However, in the image of worn burnished sample (Fig. 14), the occurrence of craters (detached surface layer) is not practically visible. The crushed surface layer occurred but without material detachment such as in the worn milled disc. On worn burnished sample cracks are visible, however, they did not lead to delamination of the surface layer. Compressive residual stresses induced by ball burnishing had a beneficial effect on crack propagation. The material of the ceramic ball was not transferred to the surface of the disc during wear. Because of the negligible wear of ball, debris originated only from the disc sample.

The ratio of the highest to the lowest wear volumes was the highest for the lowest normal load used (Fig. 12). The effect of the surface layer on wear resistance was highest for the lowest depths of the wear scar. Independently of normal load, the wear levels were the highest for milled disc samples. This performance was caused by the highest roughness and the smallest microhardness. The wear volume was also high for the burnished disc sample with the smallest pressure. This sample was characterised by low microhardness, the smallest compressive residual stresses, and the largest amplitude among burnished discs. On the other hand, the lowest volumetric wear was reached for the burnished disc with a pressure of 30 MPa. The lowest roughness height and high microhardness were the probable reasons for it. This sample also resulted in a low friction force. Burnished disc samples with pressures of 20 and 40 MPa were characterised by higher wear levels than the burnished disc sample with a pressure of 30 MPa, but smaller than the burnished disc with a pressure of 10 MPa; wear volumes of these discs were similar for normal forces of 20 and 40 N. However, for the highest normal force, less wear was achieved for the sample burnished with the highest pressure, compared to the sample machined with a burnishing pressure of 20 MPa. The higher microhardness and compressive residual stresses of a sample burnished with a pressure of 40 MPa, were the most probable reason for this behavior. Generally, a decrease in roughness height and an increase in microhardness led to an improvement in wear resistance. Wear decrease due to the increase in hardness was found in many works, for example, in^35–38^.

## Conclusions

1. Ball burnishing caused an improvement in the quality of the surface layer. Due to ball burnishing, the average surface height decreased by up to 85% and the microhardness increased by up to 20% compared to milled disc sample. Due to ball burnishing, the tensile residual stresses were transformed into compressive residual stresses. A growth in the burnishing pressure from 10 to 30 MPa caused a decrease in texture height, further pressure increase to 40 MPa caused an increase in roughness amplitude and nonhomogeneous character of surface texture. An increase in burnishing pressure caused an increase in microhardness.
2. The milled sample of the highest roughness height, of the smallest microhardness, and of the tensile residual stresses led to the highest wear levels and, in most cases, the highest coefficient of friction.
3. The beneficial tribological behaviour was obtained for the burnished disc with a pressure of 30 MPa of the lowest roughness height and high microhardness. This sample achieved the lowest wear volumes and generally led to the smallest coefficient of friction of sliding pair in the reciprocating motion. The maximum decreases in friction coefficient and wear volume compared to the milled sample were 39% and 85%, respectively.
4. The burnished disc sample with the maximum pressure of non-homogeneous surface texture, with the smallest peak density, led to a relatively great coefficient of friction.
5. High volumetric wear was obtained for the sample burnished with a pressure of 10 MPa, characterised by a large surface amplitude, small microhardness, and small compressive residual stresses. The wear volume of the burnished sample with the highest pressure of the highest microhardness was small when the maximum normal force was used.

## Data Availability

All data generated or analysed during this study are included in this publication.
